# Supplementary material for: A Humanized Yeast Phenomic Model of Deoxycytidine Kinase to Predict Genetic Buffering of Nucleoside Analog Cytotoxicity
Source: Genes (Basel). 2019 Sep 30;10(10):770. doi: 10.3390/genes10100770 (PMC6826991; doi:10.3390/genes10100770)
Supplement: Supplementary file 1 [file genes-10-00770-s001.zip › Additional_Files/Additional_File7_GOTermHeatmaps.zip!/Additional_File7_GOTermHeatmaps/snoRNA_metabolic_process.pdf]

snoRNA metabolic process

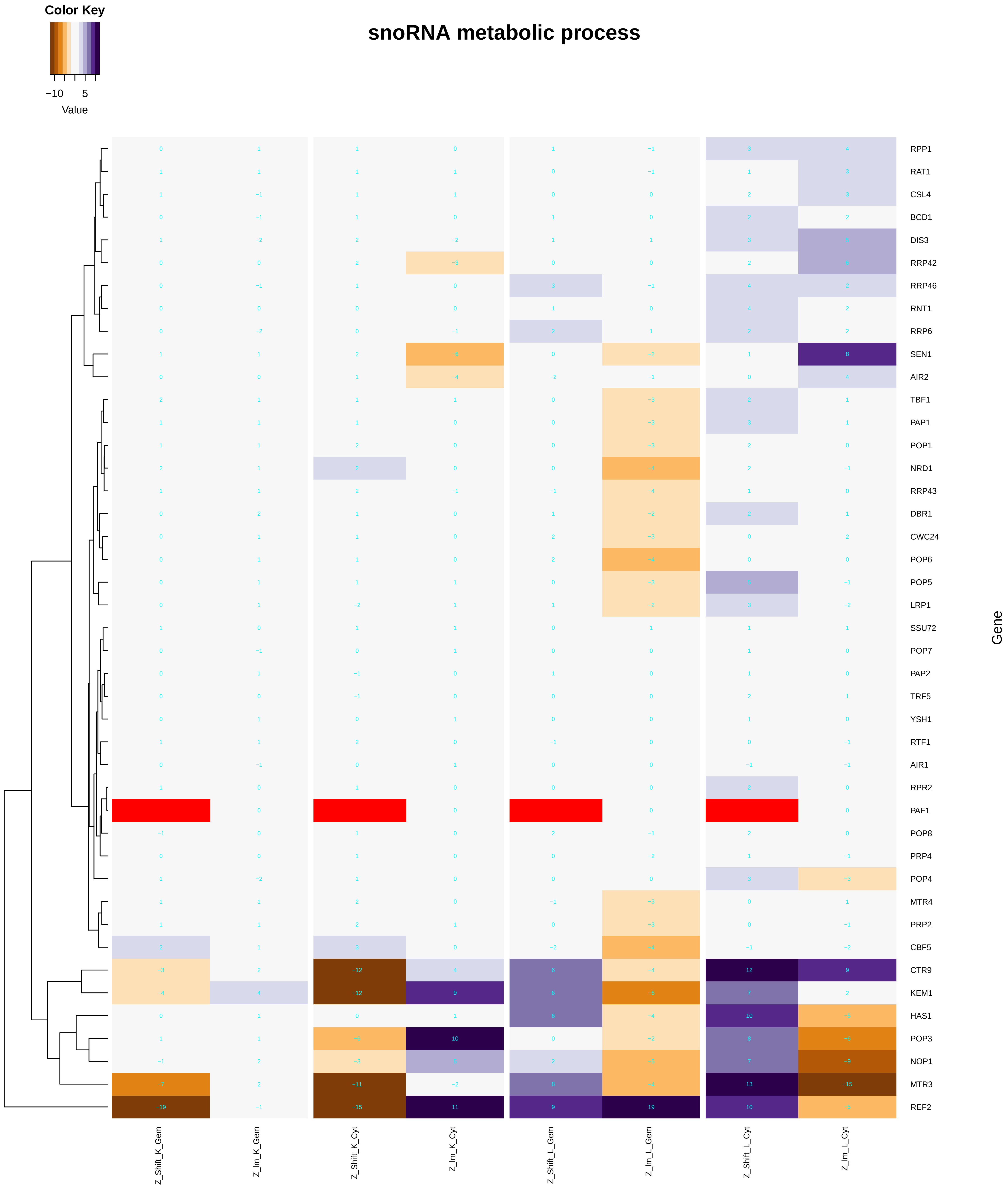

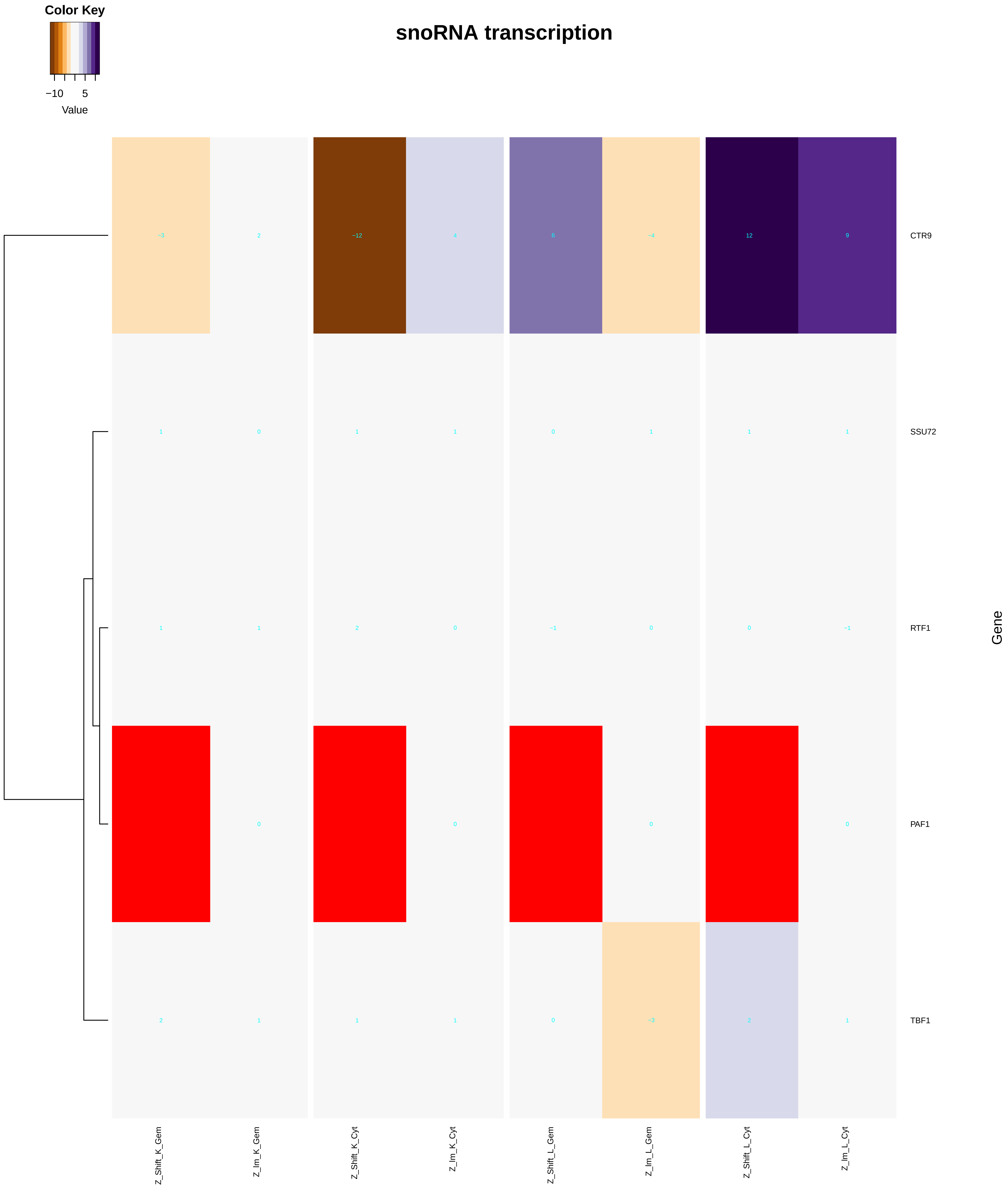
Color Key
−10
5
Value
snoRNA transcription
−3
2
−12
4
6
−4
12
9
CTR9
1
0
1
1
0
1
1
1
SSU72
1
1
2
0
−1
0
0
−1
RTF1
0
0
0
0
PAF1
2
1
1
1
0
−3
2
1
TBF1
Gene
Z_Shift_K_Gem
Z_Im_K_Gem
Z_Shift_K_Cyt
Z_Im_K_Cyt
Z_Shift_L_Gem
Z_Im_L_Gem
Z_Shift_L_Cyt
Z_Im_L_Cyt

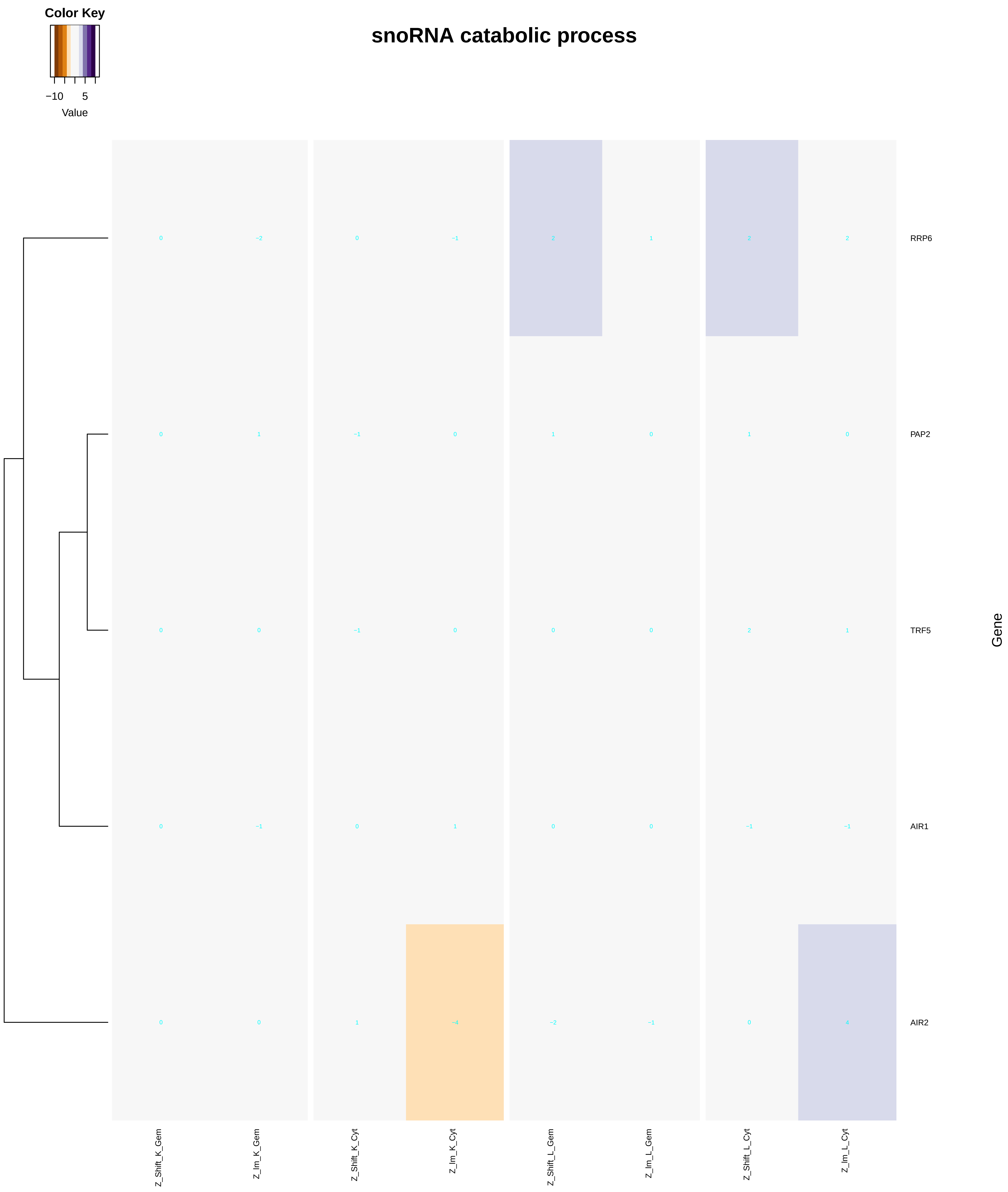

snoRNA catabolic process
Color Key
−10
5
Value
RRP6
PAP2
TRF5
AIR1
AIR2
Gene
0 −2 0 −1 2 1 2 2
0 1 −1 0 1 0 1 0
0 0 −1 0 0 0 2 1
0 −1 0 1 0 0 −1 −1
0 0 1 −4 −2 −1 0 4
Z_Shift_K_Gem
Z_Im_K_Gem
Z_Shift_K_Cyt
Z_Im_K_Cyt
Z_Shift_L_Gem
Z_Im_L_Gem
Z_Shift_L_Cyt
Z_Im_L_Cyt

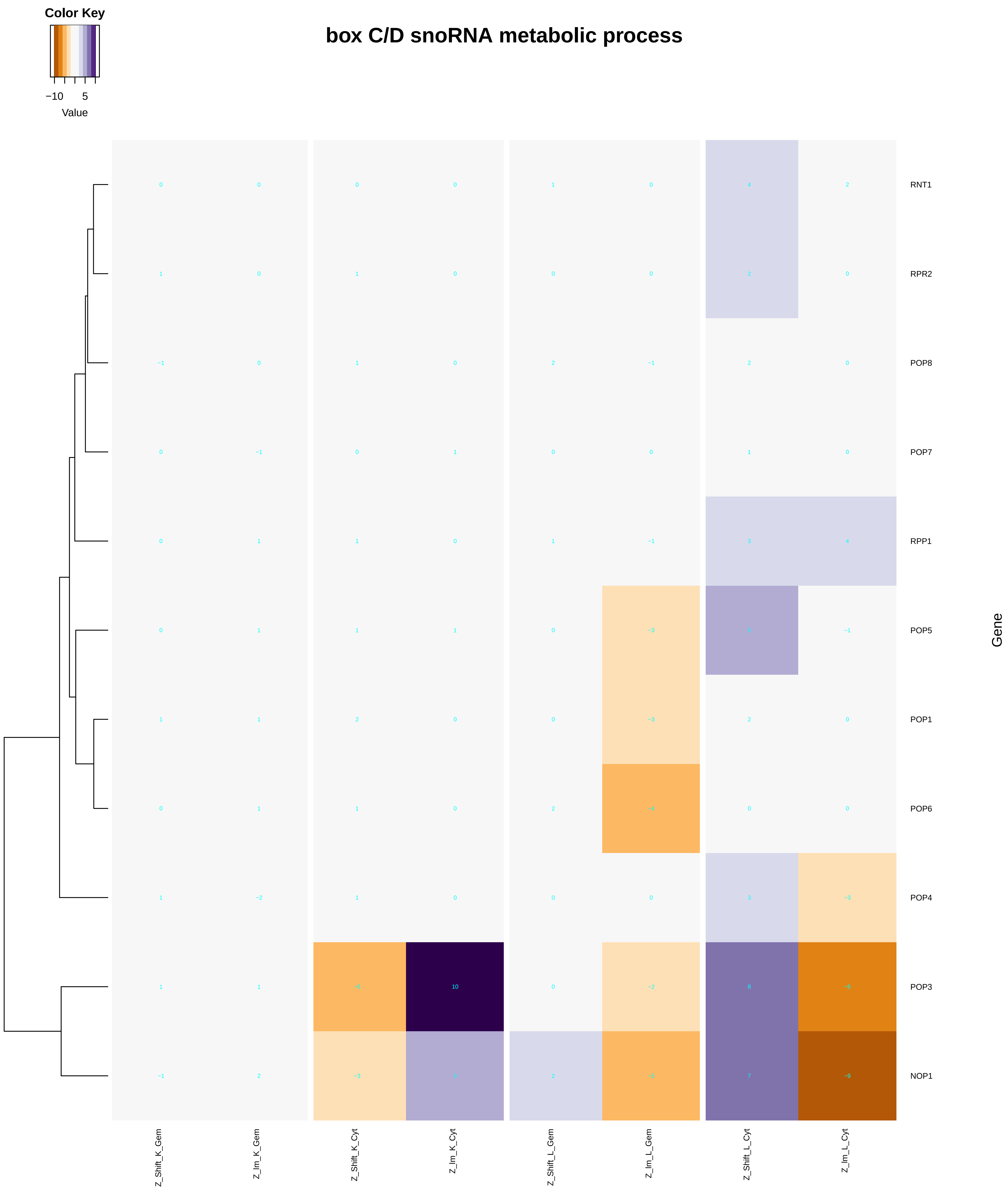
Color Key
−10
5
Value
box C/D snoRNA metabolic process
RNT1
RPR2
POP8
POP7
RPP1
POP5
POP1
POP6
POP4
POP3
NOP1
Gene
Z_Shift_K_Gem
Z_Im_K_Gem
Z_Shift_K_Cyt
Z_Im_K_Cyt
Z_Shift_L_Gem
Z_Im_L_Gem
Z_Shift_L_Cyt
Z_Im_L_Cyt

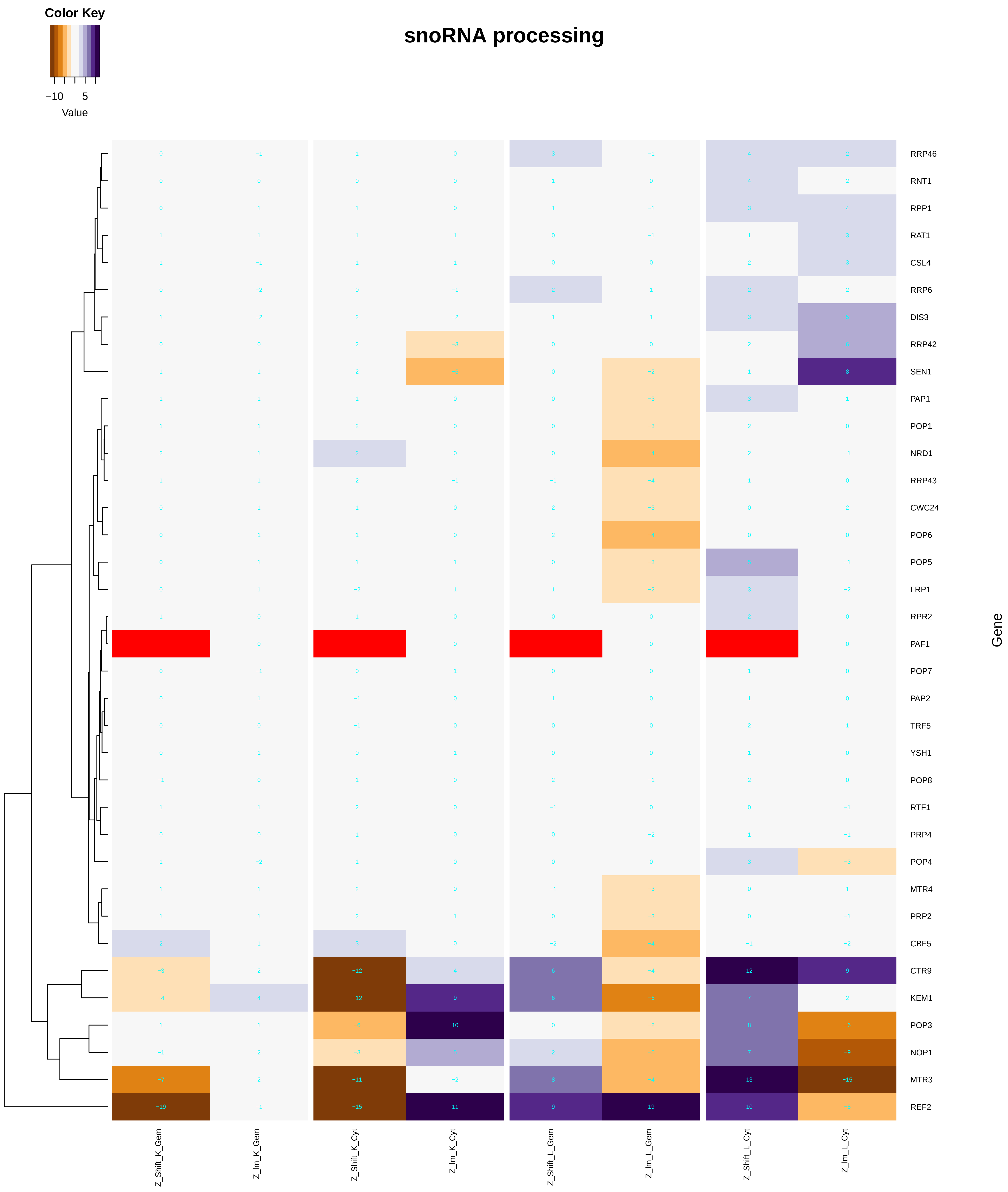

Color Key
−10
5
Value
snoRNA processing
RRP46
RNT1
RPP1
RAT1
CSL4
RRP6
DIS3
RRP42
SEN1
PAP1
POP1
NRD1
RRP43
CWC24
POP6
POP5
LRP1
RPR2
PAF1
POP7
PAP2
TRF5
YSH1
POP8
RTF1
PRP4
POP4
MTR4
PRP2
CBF5
CTR9
KEM1
POP3
NOP1
MTR3
REF2
Gene
Z_Shift_K_Gem
Z_Im_K_Gem
Z_Shift_K_Cyt
Z_Im_K_Cyt
Z_Shift_L_Gem
Z_Im_L_Gem
Z_Shift_L_Cyt
Z_Im_L_Cyt

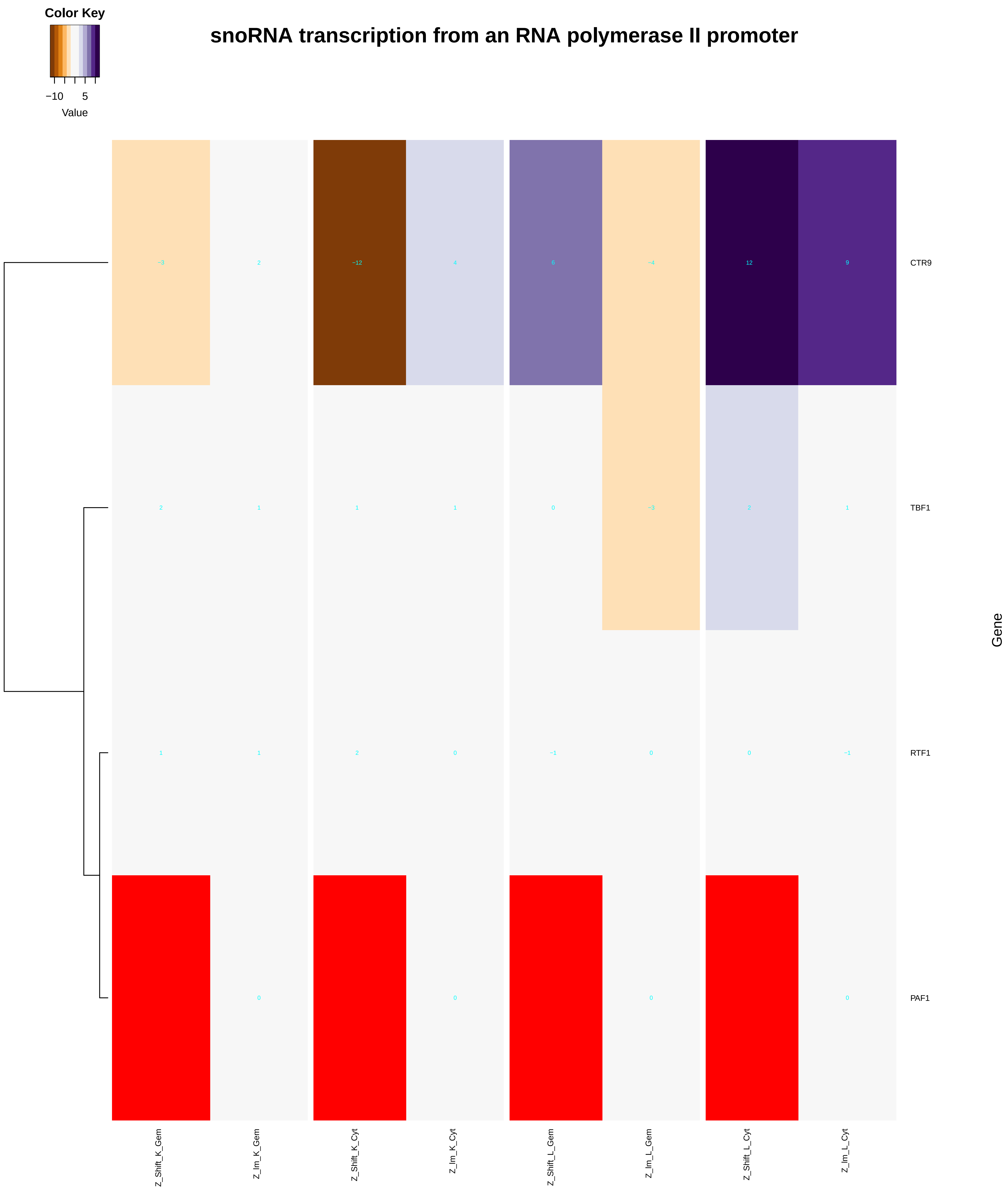

Color Key
−10
5
Value
snoRNA transcription from an RNA polymerase II promoter
CTR9
TBF1
RTF1
PAF1
Gene
Z_Shift_K_Gem
Z_Im_K_Gem
Z_Shift_K_Cyt
Z_Im_K_Cyt
Z_Shift_L_Gem
Z_Im_L_Gem
Z_Shift_L_Cyt
Z_Im_L_Cyt

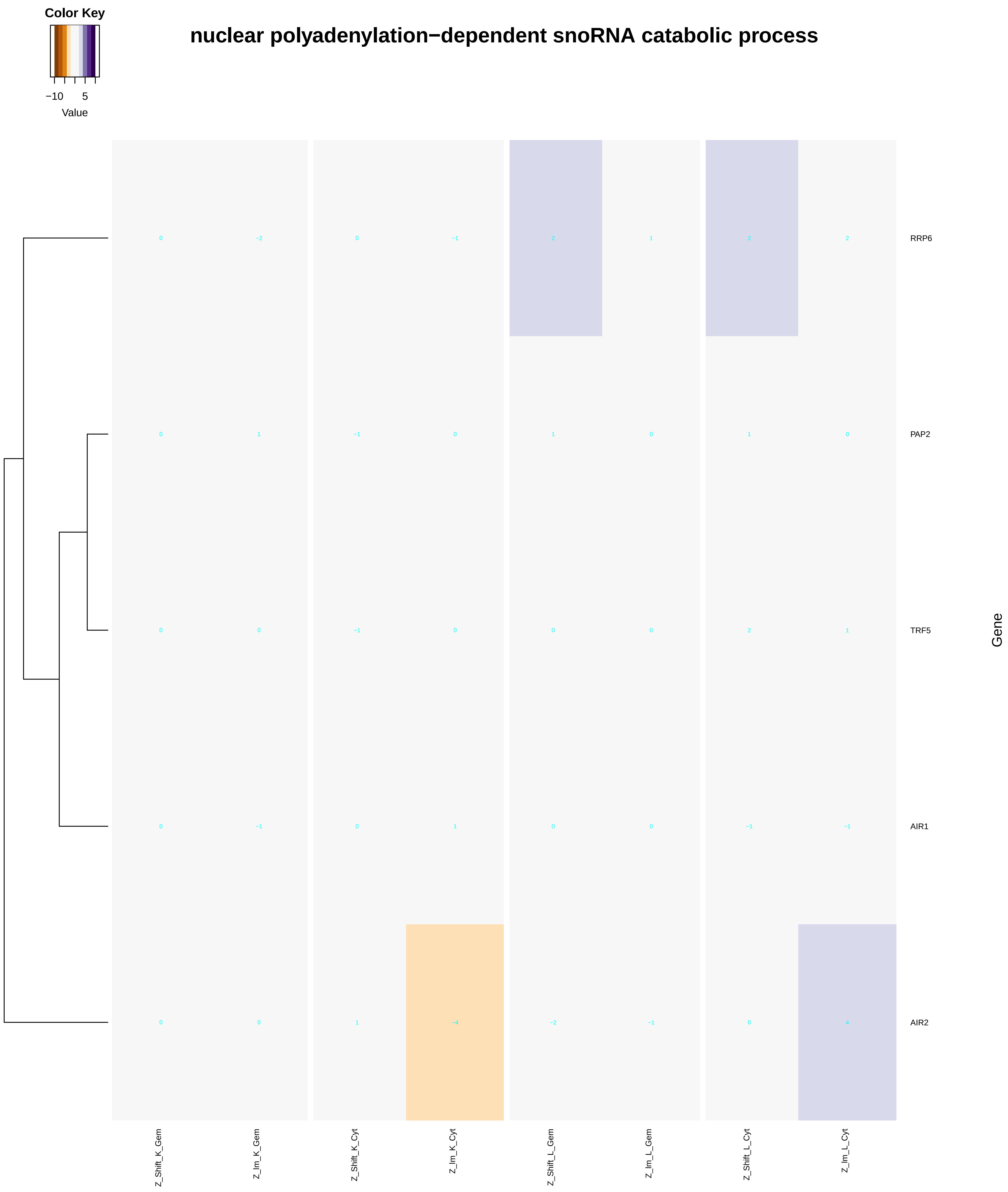
Color Key
−10
5
Value
nuclear polyadenylation−dependent snoRNA catabolic process
RRP6
PAP2
TRF5
AIR1
AIR2
Gene
Z_Shift_K_Gem
Z_Im_K_Gem
Z_Shift_K_Cyt
Z_Im_K_Cyt
Z_Shift_L_Gem
Z_Im_L_Gem
Z_Shift_L_Cyt
Z_Im_L_Cyt

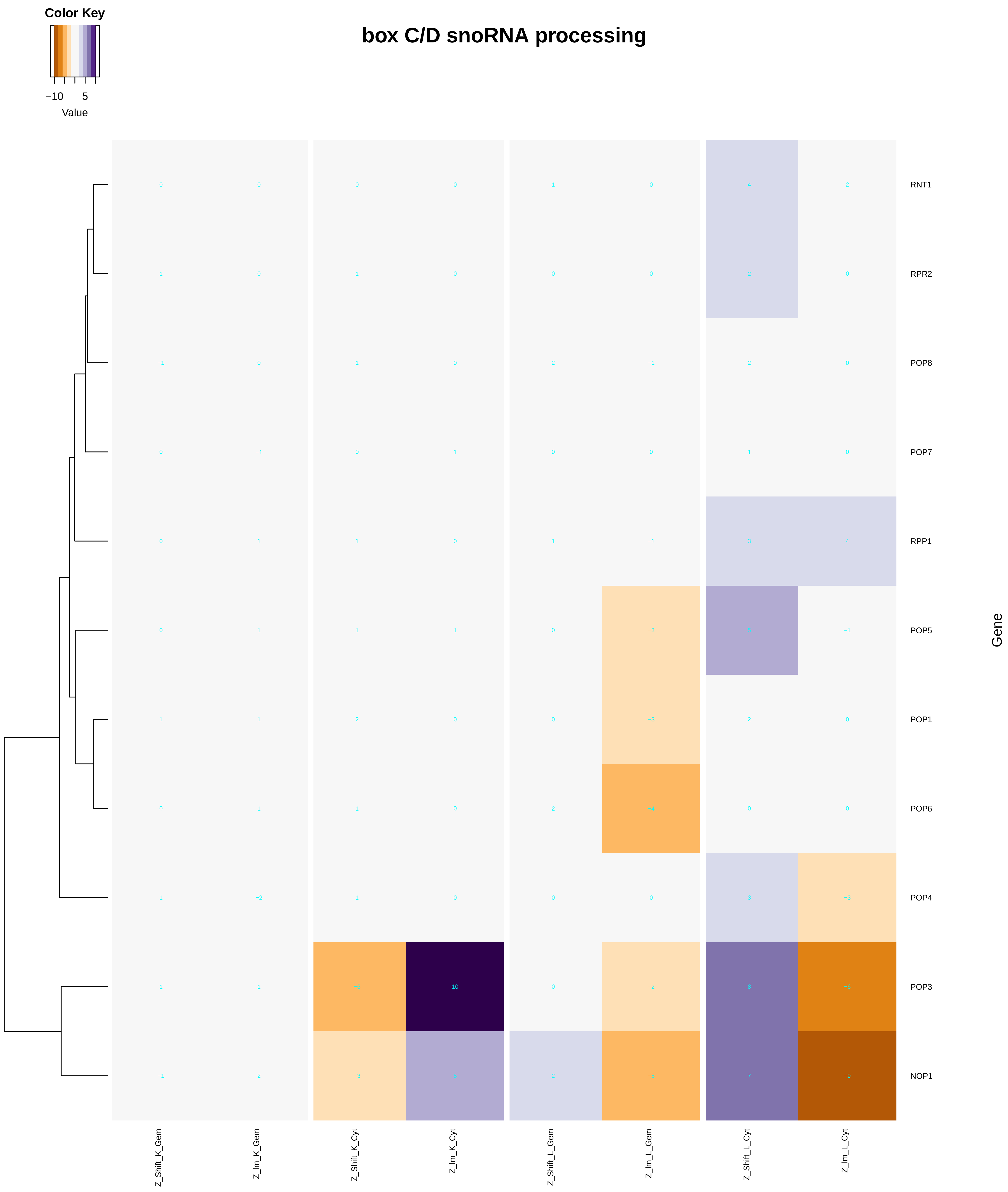
Color Key
−10
5
Value
box C/D snoRNA processing
RNT1
RPR2
POP8
POP7
RPP1
POP5
POP1
POP6
POP4
POP3
NOP1
Gene
Z_Shift_K_Gem
Z_Im_K_Gem
Z_Shift_K_Cyt
Z_Im_K_Cyt
Z_Shift_L_Gem
Z_Im_L_Gem
Z_Shift_L_Cyt
Z_Im_L_Cyt

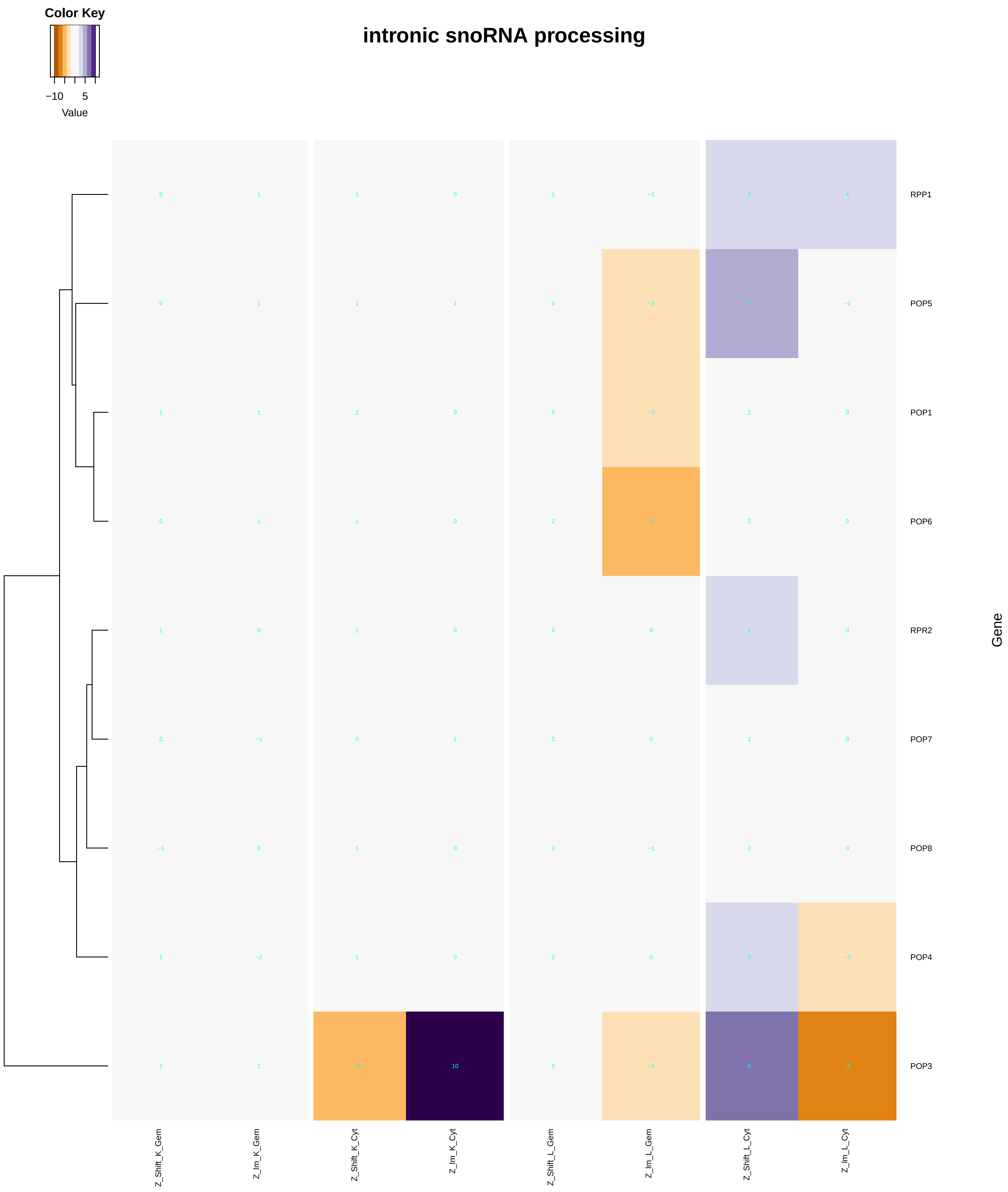
Color Key
−10
5
Value
intronic snoRNA processing
RPP1
POP5
POP1
POP6
RPR2
POP7
POP8
POP4
POP3
Gene
Z_Shift_K_Gem
Z_Im_K_Gem
Z_Shift_K_Cyt
Z_Im_K_Cyt
Z_Shift_L_Gem
Z_Im_L_Gem
Z_Shift_L_Cyt
Z_Im_L_Cyt

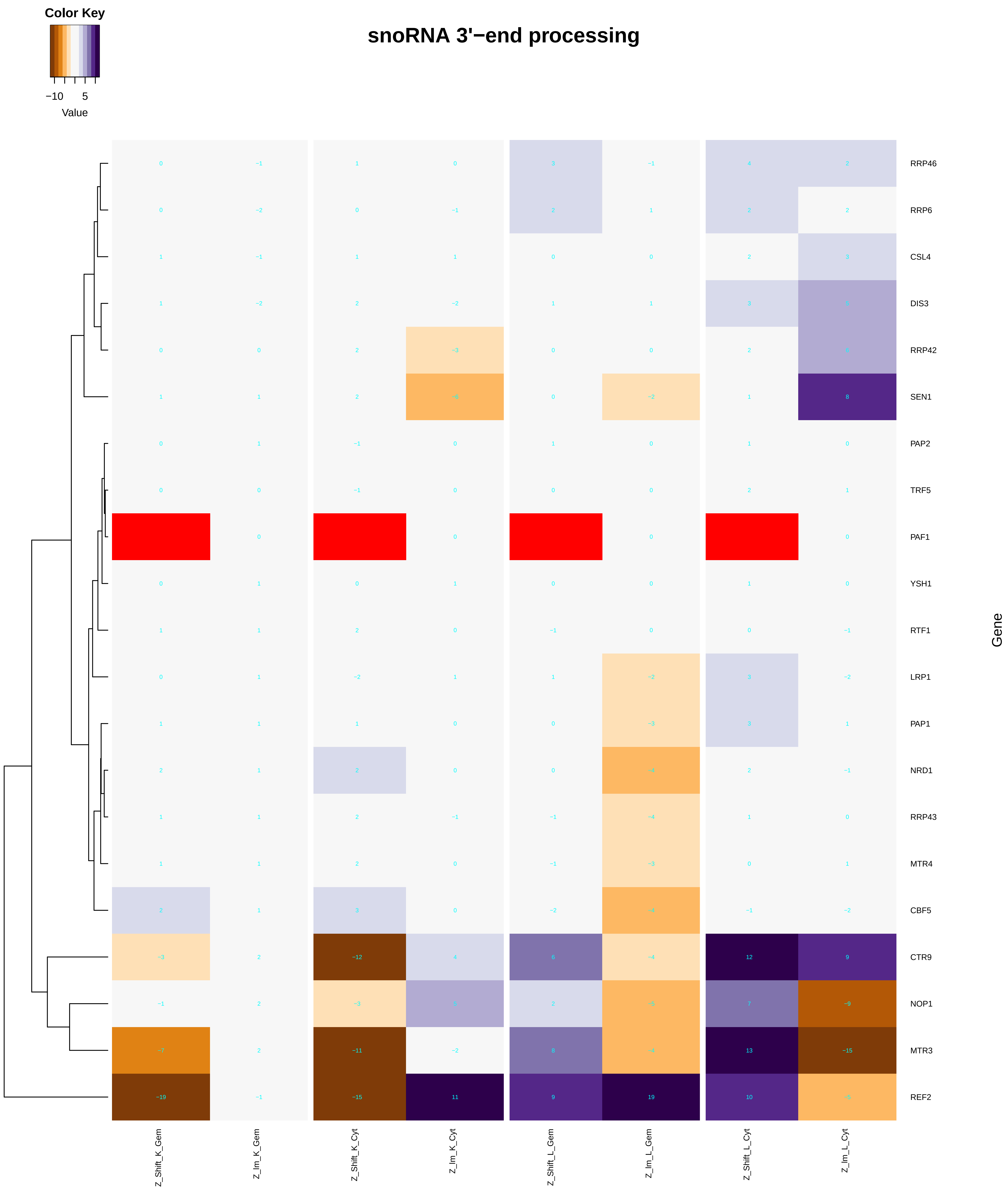

Color Key
−10 5
Value
snoRNA 3'−end processing
RRP46
RRP6
CSL4
DIS3
RRP42
SEN1
PAP2
TRF5
PAF1
YSH1
RTF1
LRP1
PAP1
NRD1
RRP43
MTR4
CBF5
CTR9
NOP1
MTR3
REF2
Gene
Z_Shift_K_Gem
Z_Im_K_Gem
Z_Shift_K_Cyt
Z_Im_K_Cyt
Z_Shift_L_Gem
Z_Im_L_Gem
Z_Shift_L_Cyt
Z_Im_L_Cyt

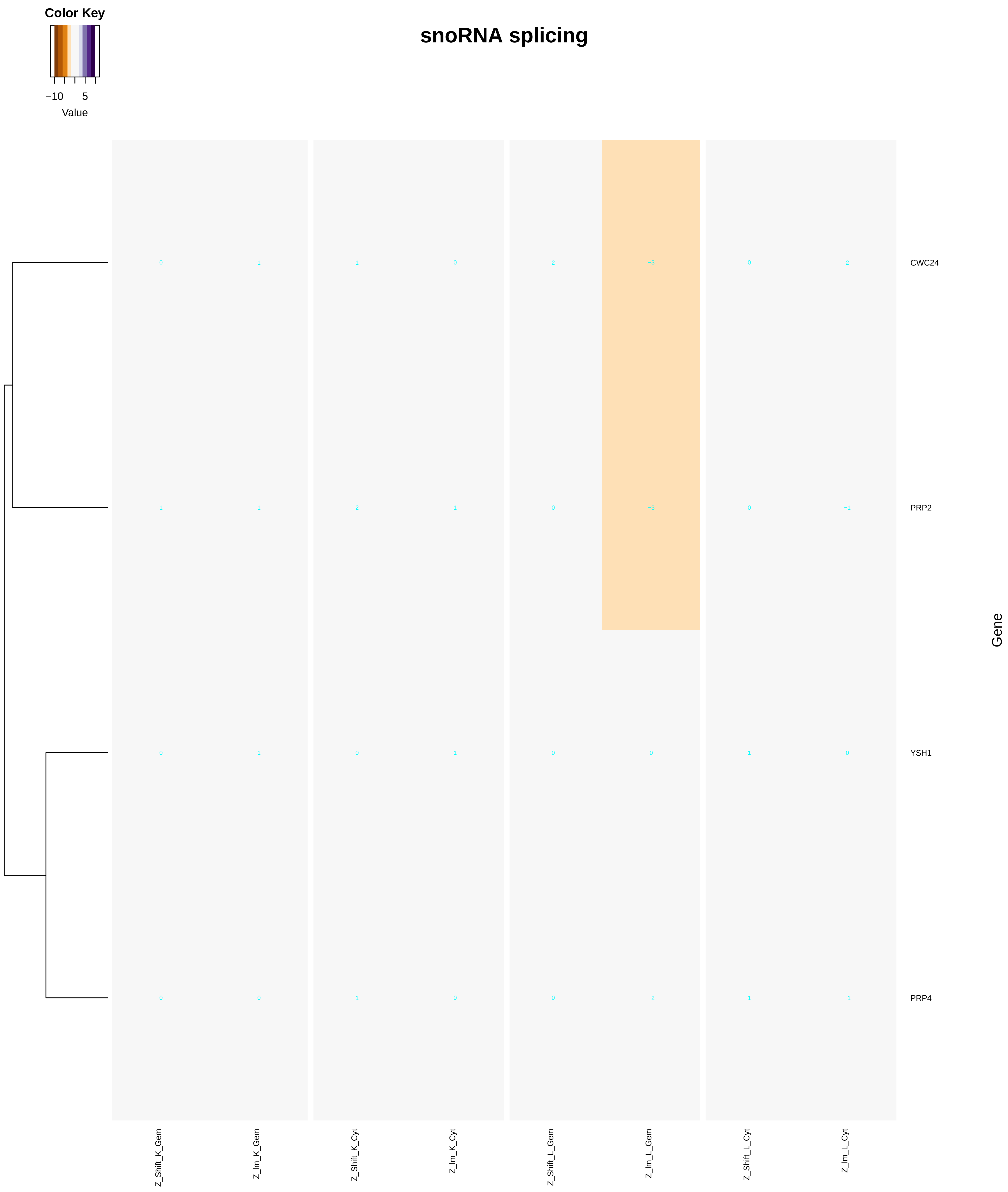

Color Key
−10
5
Value
snoRNA splicing
CWC24
PRP2
YSH1
PRP4
Gene
Z_Shift_K_Gem
Z_Im_K_Gem
Z_Shift_K_Cyt
Z_Im_K_Cyt
Z_Shift_L_Gem
Z_Im_L_Gem
Z_Shift_L_Cyt
Z_Im_L_Cyt

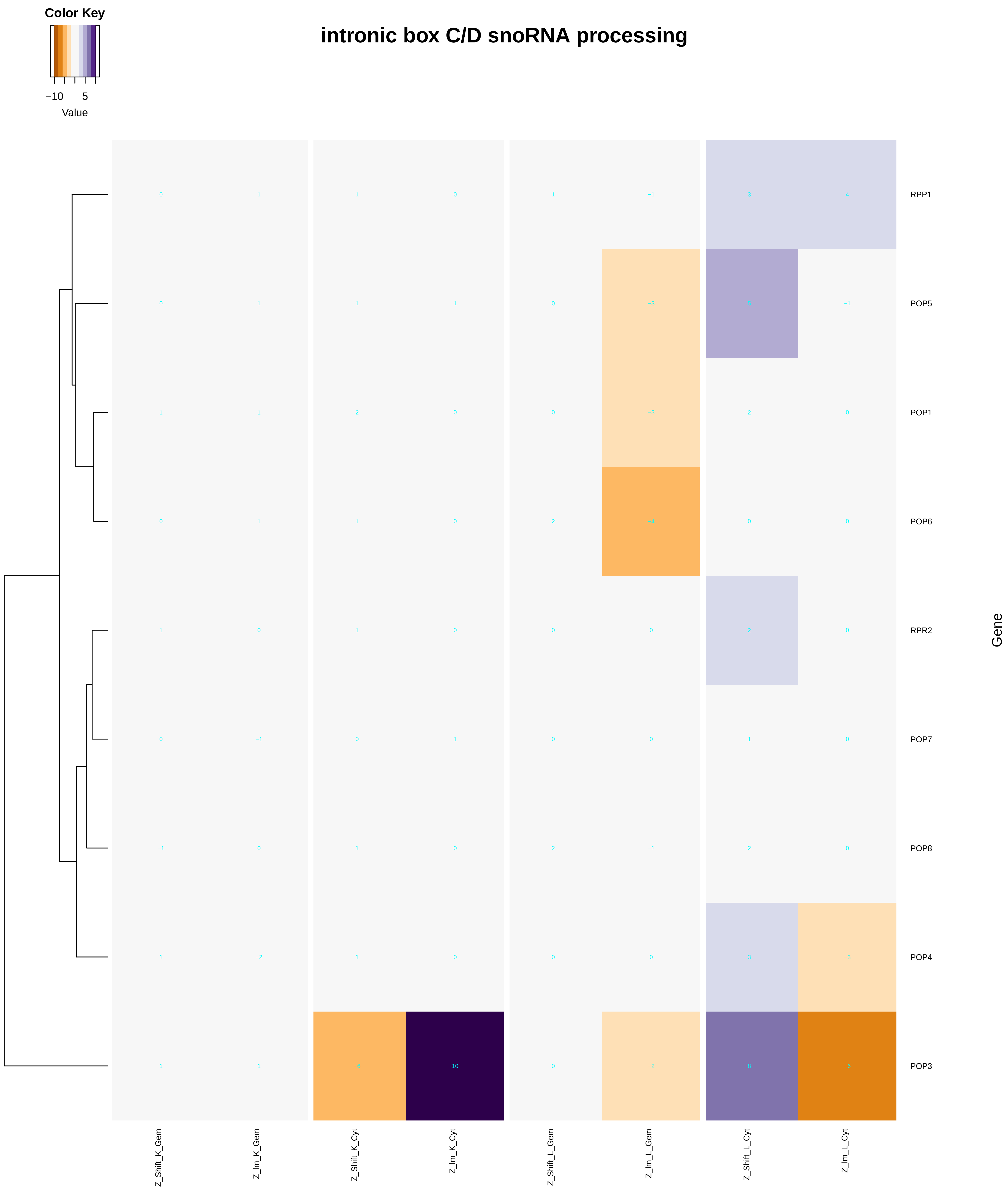

intronic box C/D snoRNA processing
Color Key
−10
5
Value
RPP1
POP5
POP1
POP6
RPR2
POP7
POP8
POP4
POP3
Gene
Z_Shift_K_Gem
Z_Im_K_Gem
Z_Shift_K_Cyt
Z_Im_K_Cyt
Z_Shift_L_Gem
Z_Im_L_Gem
Z_Shift_L_Cyt
Z_Im_L_Cyt

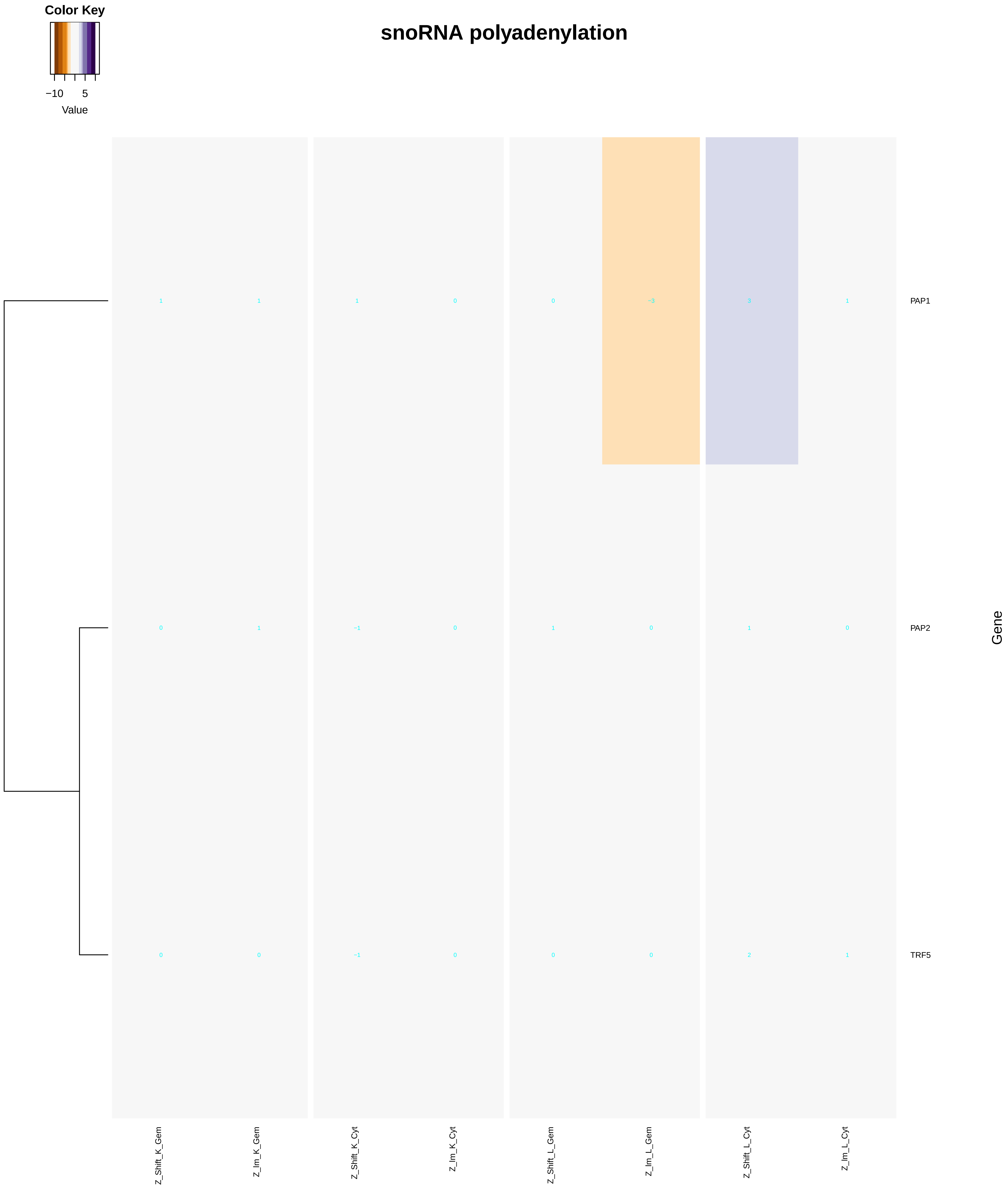
Color Key
−10
5
Value
snoRNA polyadenylation
1 1 1 0 0 −3 3 1
PAP1
0 1 −1 0 1 0 1 0
PAP2
0 0 −1 0 0 0 2 1
TRF5
Gene
Z_Shift_K_Gem
Z_Im_K_Gem
Z_Shift_K_Cyt
Z_Im_K_Cyt
Z_Shift_L_Gem
Z_Im_L_Gem
Z_Shift_L_Cyt
Z_Im_L_Cyt

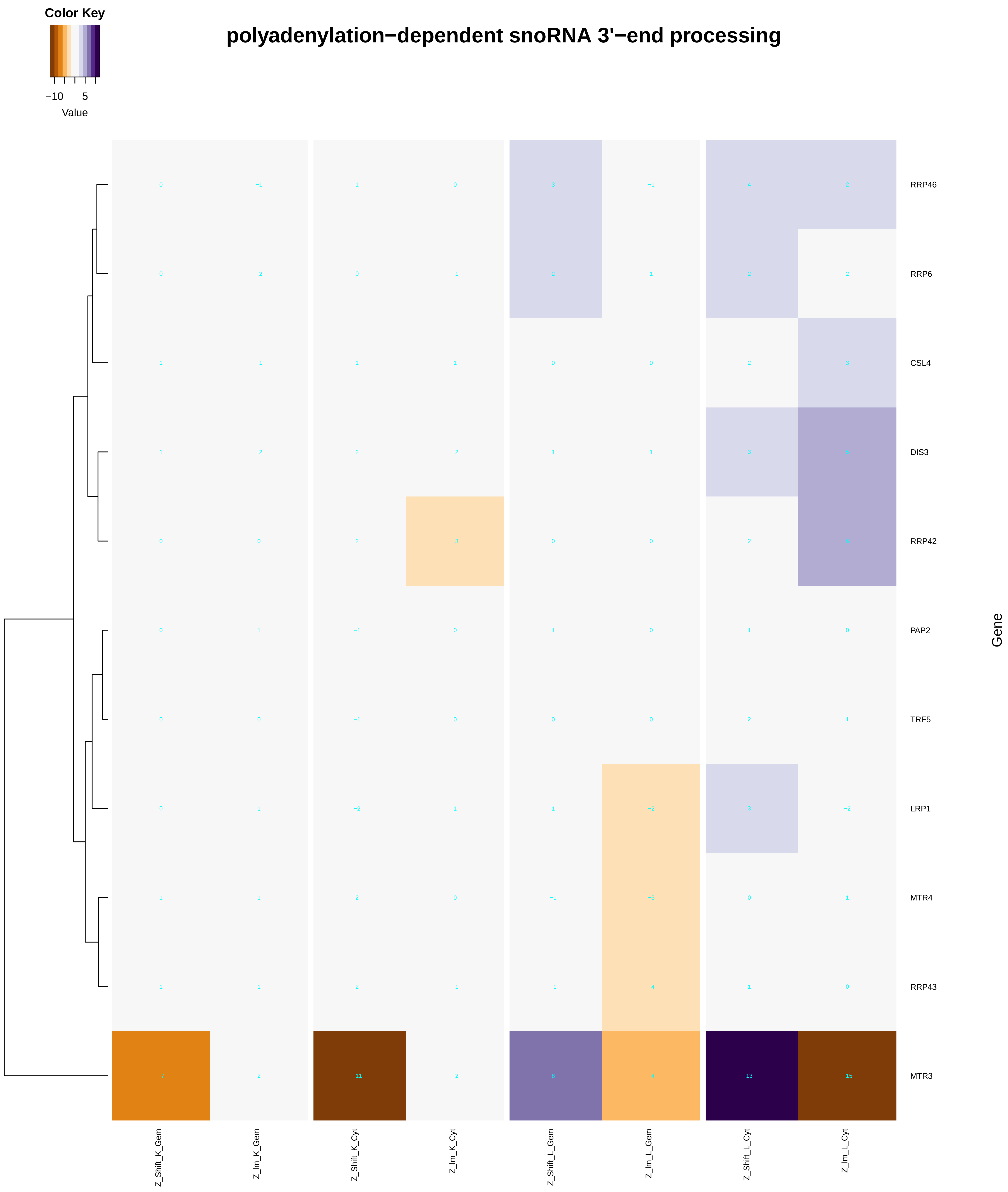
Color Key
−10
5
Value
polyadenylation−dependent snoRNA 3'−end processing
RRP46
RRP6
CSL4
DIS3
RRP42
PAP2
TRF5
LRP1
MTR4
RRP43
MTR3
Gene
Z_Shift_K_Gem
Z_Im_K_Gem
Z_Shift_K_Cyt
Z_Im_K_Cyt
Z_Shift_L_Gem
Z_Im_L_Gem
Z_Shift_L_Cyt
Z_Im_L_Cyt
